# Hemi-Gastrocnemius Hemi-Soleus Bi-pedicled Muscle Flap and Bone Transport in Reconstruction of Bone and Soft Tissue Defects in an Open Fracture Tibia Gustilo Type IIIc

**DOI:** 10.7759/cureus.75294

**Published:** 2024-12-07

**Authors:** Mohammad A Ali, Mohammed A Sanad, Mohamed O Elhassan, Khalid Alawadi

**Affiliations:** 1 Hand and Microsurgery Unit, Trauma and Orthopedic Department, Rashid Hospital, Dubai Health Authority, Dubai, ARE; 2 Orthopedics and Traumatology, Rashid Hospital, Dubai Health Authority, Dubai, ARE; 3 Orthopedics and Traumatology, Medcare Orthopedics and Spine Hospital, Dubai Health Authority, Dubai, ARE

**Keywords:** bi-pedicle muscle flap, hemigastrocnemus hemisoleus, local flap, open fracture tibia, reconstruction

## Abstract

Soft tissue injury in open fracture of the lower extremity represents a challenging trauma that requires complex strategies to reconstruct both bony and soft tissue defects. Various options are available to cover the soft tissue defect in the lower extremities, from simple skin grafting to local fasciocutaneous and muscle flaps. However, when the injury is extensive and involves a large surface area, options for treatment of local flap coverage become limited. Vascular injury (Gustilo type IIIc) further increases the risk of complications and flap failure. Despite these complexities, meticulous planning and attention to detail can optimize patient outcomes. Herein, we present a case of an open lower extremity fracture involving a substantial soft tissue defect over the proximal and middle segments of the tibia complicated by a vascular injury (Gustilo type IIIc). A combination of the hemigastrocnemius and hemisoleus muscles was implemented to cover soft tissue defect over the proximal two-thirds of the tibia despite significant popliteal artery compromise. This case underscores the feasibility of using a combined local muscle flap approach involving the hemigastrocnemius and hemisoleus muscles in managing open fractures with concomitant vascular injuries.

## Introduction

Open fracture of the tibia complicated by a large soft tissue defect, bone loss, and vascular compromise presents a challenging injury for both reconstructive surgeons. The goal of soft tissue reconstruction in lower extremity injuries is to achieve reliable and early soft tissue coverage [1]. Management of such cases requires radical debridement, suitable bone fixation according to the fracture pattern, and early flap coverage to avoid complications such as bone necrosis and infections [2]. Many types of flaps can be implemented in such cases, ranging from simple local flaps to free microvascular flaps [3]. The proper flap selection depends on the viability of the surrounding tissue, the availability of the microsurgery setup, and the patient’s general condition. Reddy and Stevenson discussed the traditional zones of the leg wherein regional muscle flaps are used in the proximal two-thirds, traditional free flaps are used in the distal third, and muscle flaps are used in the foot [4]. However, with extensive soft tissue injury that involves more than one zone or with concomitant vascular injury, as in Gustilo grade IIIC open fracture, it is often found that the pedicle of these local tissue flaps lies within the zone of injury precluding their use [5]. Due to our improved understanding of the vascular anatomy of the lower extremity and the vascular territory along with the improved investigational tools such as CT angiography, it is now increasingly possible to carefully plan and execute local flaps even in the presence of more extensive soft tissue injury [6-9].

Here, we present a case of an open Gustilo IIIC leg fracture with a large soft tissue defect over the proximal two-thirds of the tibia and popliteal artery injury, treated with combined hemigastrocnemius and hemisoleus local muscle flaps after preoperative assessment of the patency and flow in the sural artery using CT angiography. This case highlights the validity and reliability of the local flap as a simple solution for complex injury if carefully planned.

## Case presentation

**Figure 1:**
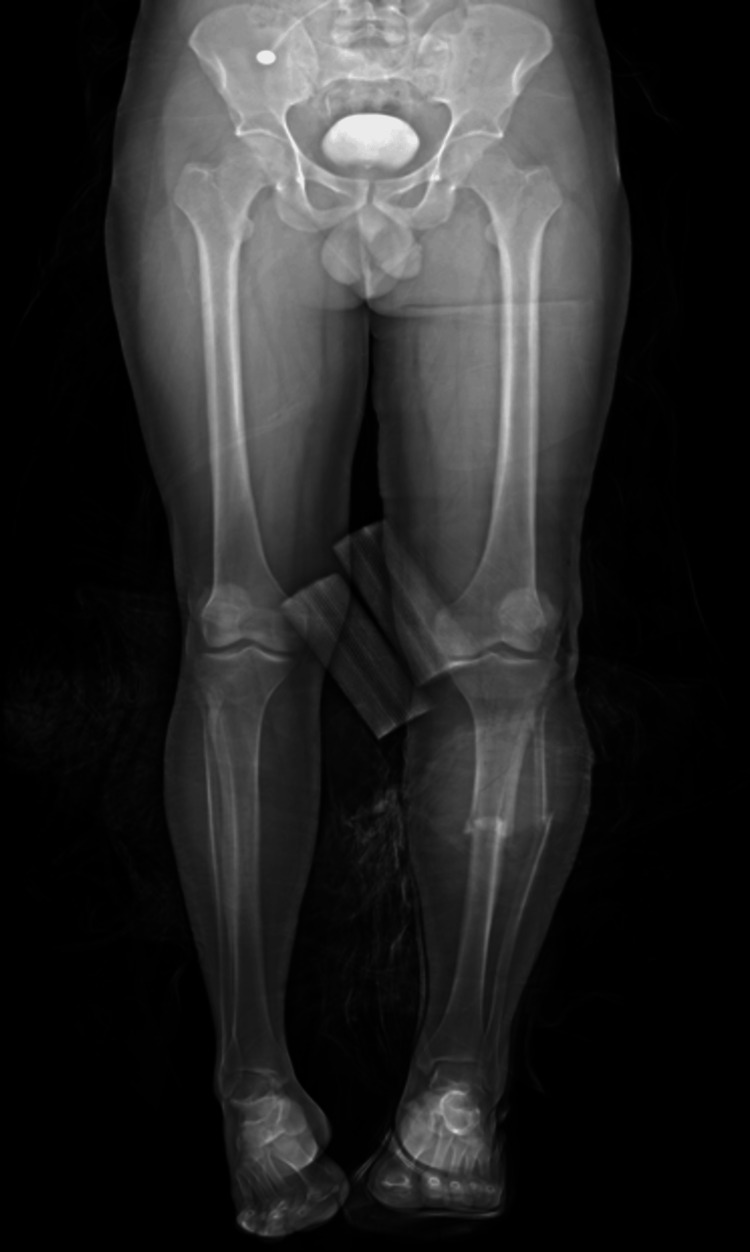
X-ray of the lower limb revealed a fracture of the left tibia.

**Figure 2:**
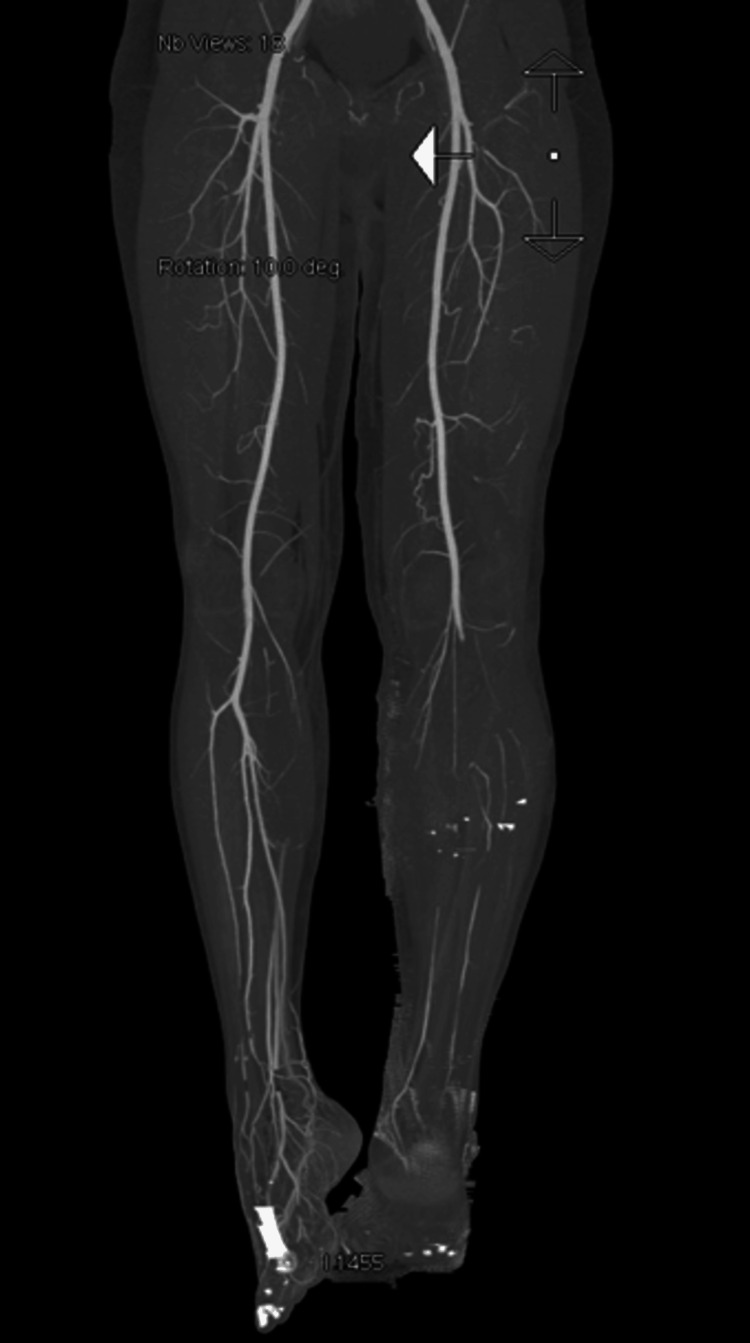
CT angiography revealed a popliteal artery injury, and intact medial and lateral sural artery supplying the gastrocnemius muscles.

A 34-year-old male patient presented to the emergency department after a motor vehicle accident caused by a rear-end collision. On arrival, the patient was alert, conscious, and oriented, with a Glasgow Coma Scale (GCS) score of 15/15. Physical examination revealed absent distal pedal pulses in the leg and no detectable Doppler signals. Two lacerated wounds were observed on the anterior surface of the proximal third of the leg and on the anterior aspect of the middle third of the leg, with active bleeding. Radiographic imaging via a stat X-ray revealed an open fracture of the tibia at the junction between the upper and middle thirds, with the fracture line extending to the proximal third of the tibia and a segmented fracture of the fibula (Figure 1). CT angiography showed a popliteal artery injury (Figure 2).

**Figure 3:**
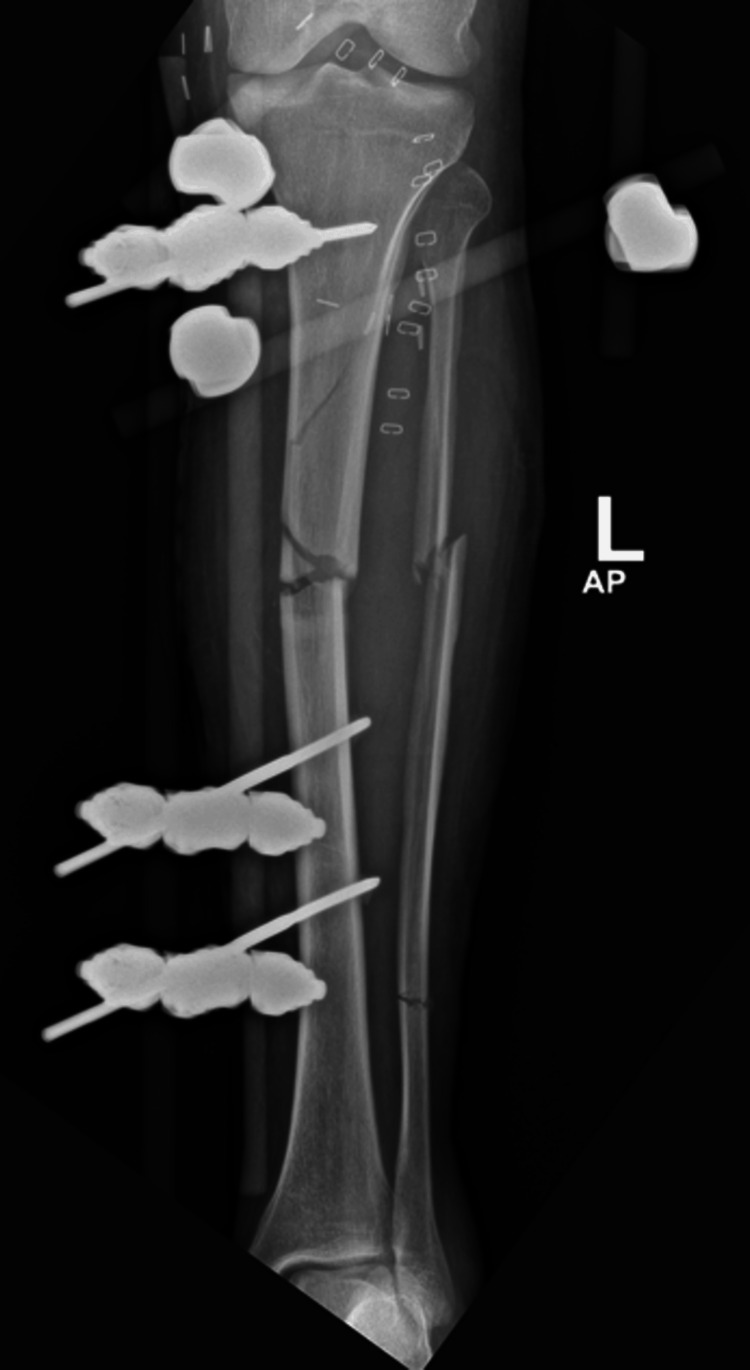
Postoperative anteroposterior radiograph of the left tibia and fibula showing an external fixator in place. The tibia demonstrated a comminuted fracture with a medial butterfly fragment and a fracture line extending to the proximal third. The fibula exhibits a segmental fracture.

The patient was immediately transferred to the operating room for a multidisciplinary team approach. The vascular team performed an urgent exploration that revealed a large, thrombosed segment in the distal femoral and popliteal arteries. The thrombosed segment was resected, and the artery was repaired using a venous graft. The orthopedic team debrided all necrotic and unhealthy tissue in the upper and middle thirds of the leg. Medial and lateral fasciotomy wounds were created to release the leg compartments, and a uniplanar external fixator was applied (Figure 3).

**Figure 4:**
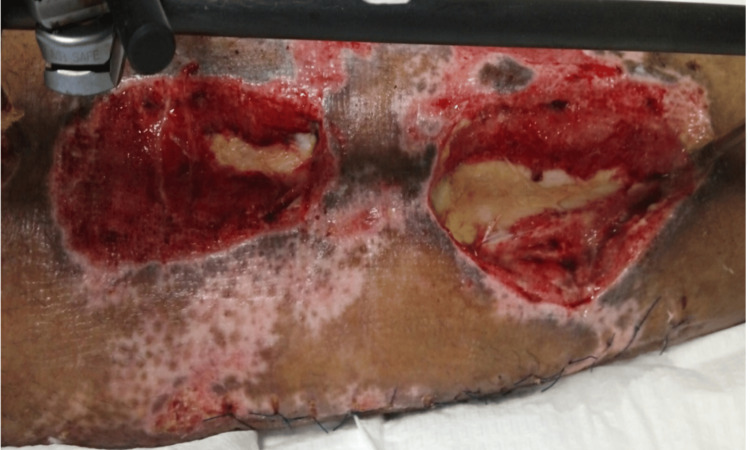
Postoperative photograph of the left leg, demonstrating the exposed and nonviable bone segment in the middle third of the tibia.

On postoperative day one, the leg became cold and pulseless, showing signs of ischemia, and a Doppler ultrasound indicated decreased blood flow in the arteries. The patient underwent an immediate exploration, which revealed thrombosis of the venous graft and impaired flow in the femoral and popliteal arteries again. The thrombosed venous graft was excised, and a new venous graft was harvested and anastomosed. Following anastomosis, satisfactory flow and distal circulation were found. During follow-up, there were no signs of infection, and vascularity to the distal part of the leg and foot remained intact. However, the bone in the middle third of the tibia was deemed nonviable (Figure 4).

Following five rounds of wound debridement and negative pressure therapy, the fasciotomy wounds were closed. A segment of necrotic bone, approximately 11 cm long, was removed from the middle third of the tibia. This left two large soft tissue defects, measuring 5x6 cm and 8x7 cm, respectively, over the upper and middle thirds of the tibia.

**Figure 5:**
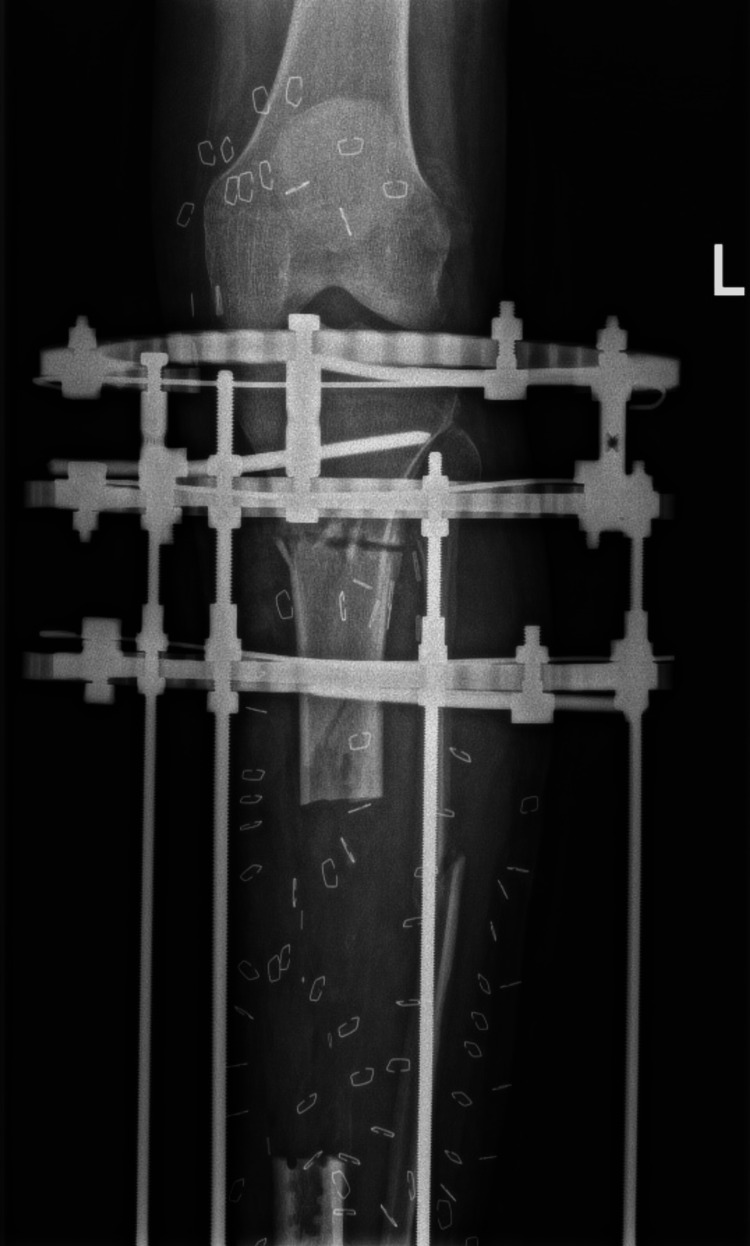
A radiograph of the left tibia showing a corticotomy and lengthening over the Ilizarov frame

**Figure 6:**
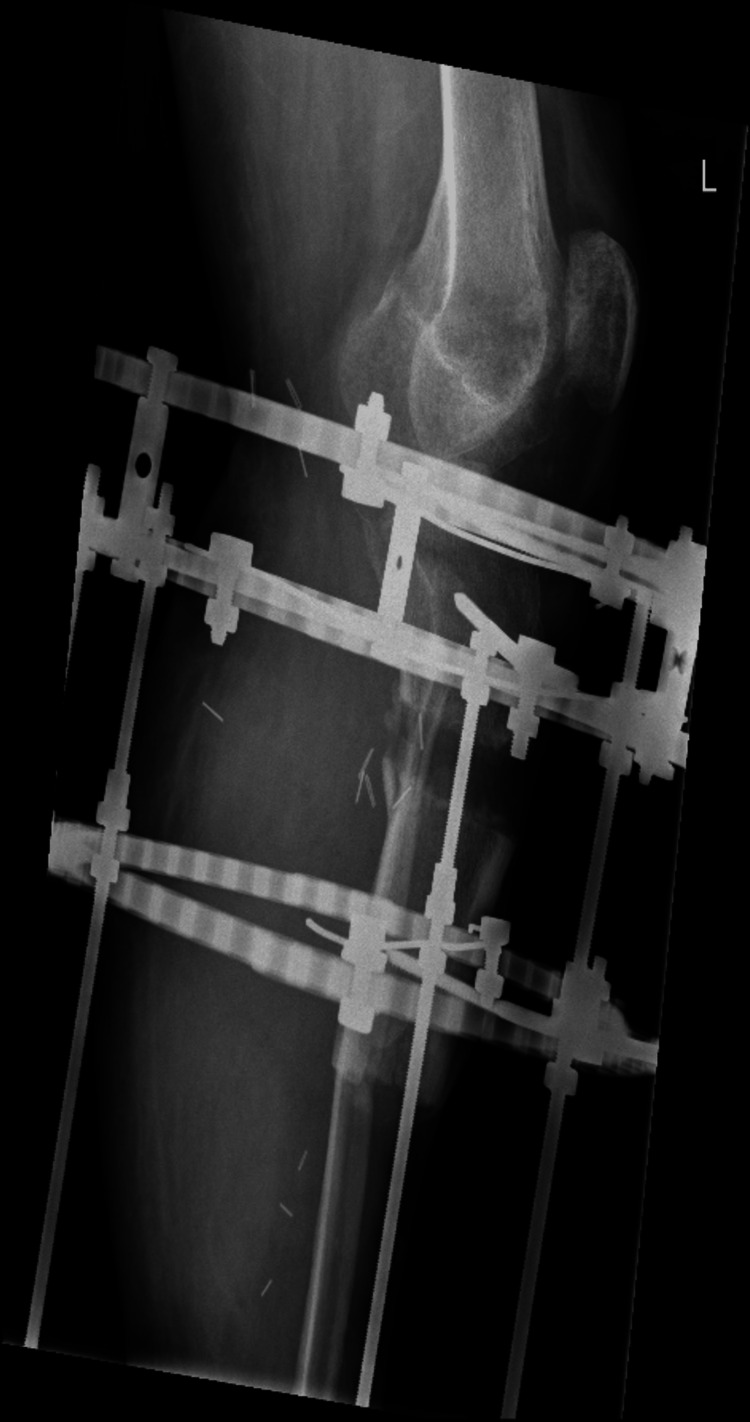
A radiograph of the left tibia showing a corticotomy and lengthening over the Ilizarov frame

**Figure 7:**
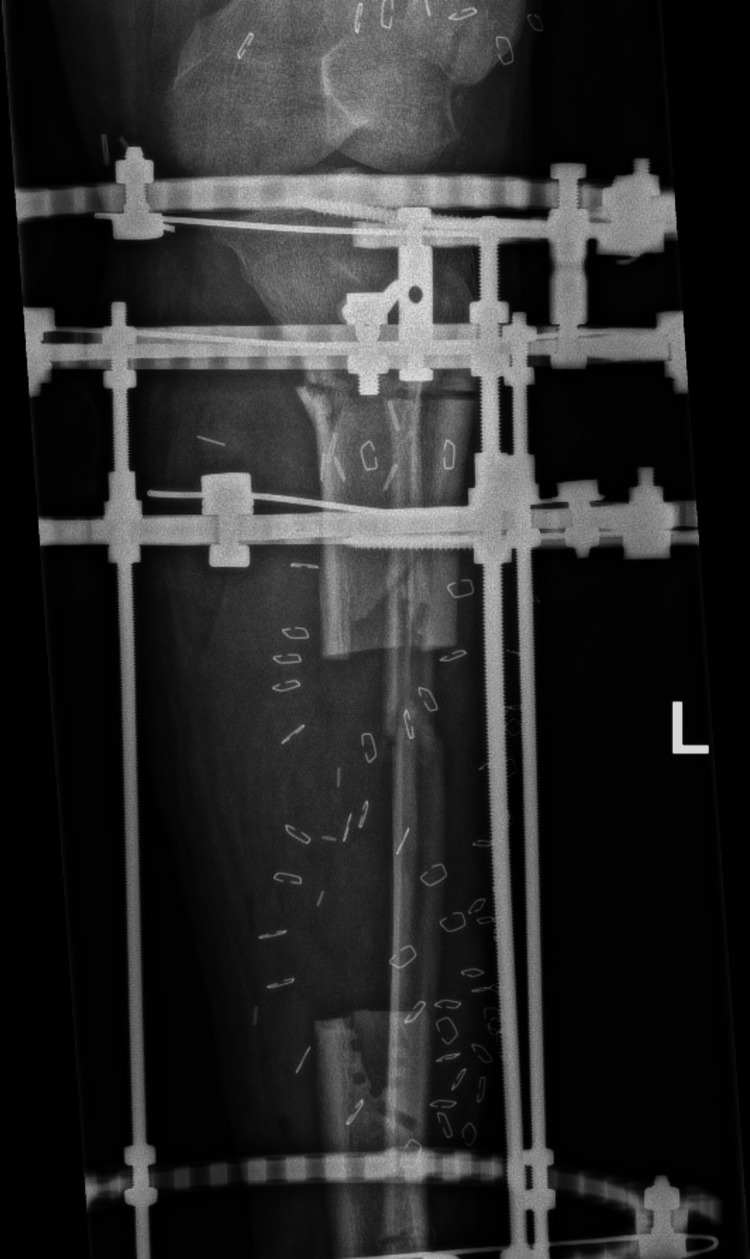
A radiograph of the left tibia showing a corticotomy and lengthening over the Ilizarov frame

CT angiography performed prior to the final surgery revealed an intact sural artery branch from the popliteal artery with laminar blood flow in the medial and lateral branches. This finding altered the surgical approach from a free flap for soft-tissue coverage to a combined medial hemigastrocnemius and hemisoleus local muscle flap. During the final surgery, an Ilizarov frame was applied to the tibia with a proximal tibial corticotomy for future bone transport (Figure 5-7).

**Figure 8:**
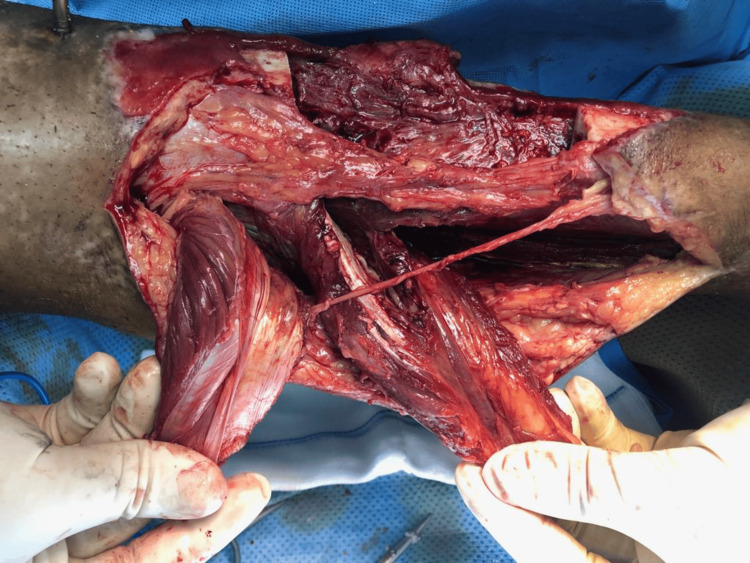
A photograph of the proximal-based hemigastrocnemius and hemisoleus flaps, demonstrating healthy and viable tissue.

**Figure 9:**
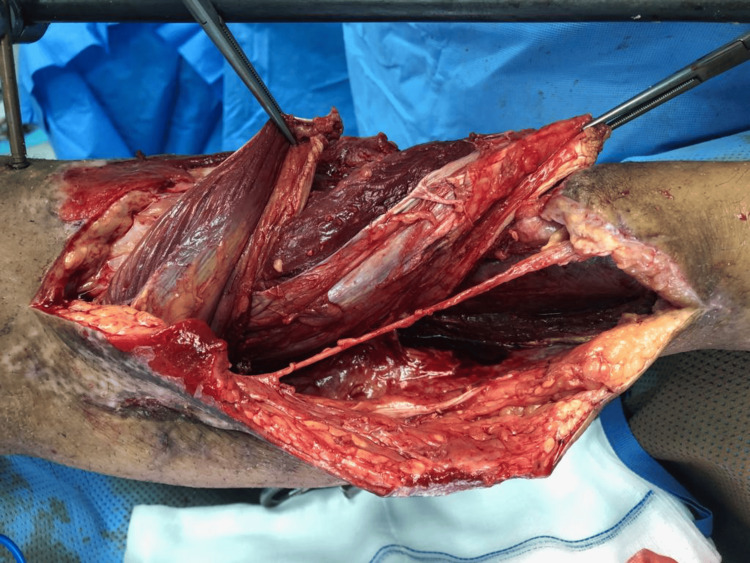
A photograph of the proximal-based hemigastrocnemius and hemisoleus flaps, demonstrating healthy and viable tissue.

The soft tissue was covered as planned with a combined medial hemigastrocnemuis and hemisoleous local muscles flap (Figure 8-9).

**Figure 10:**
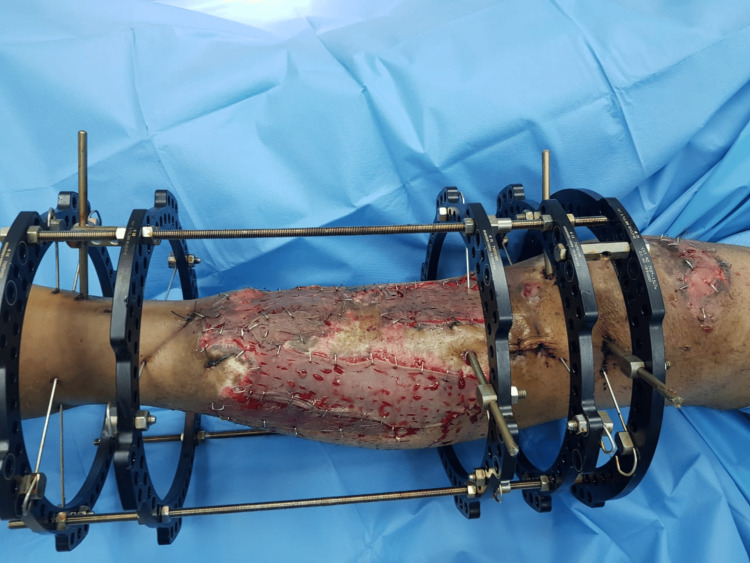
A photograph of the left leg showing an exposed area on the lateral aspect and a muscle flap on the medial aspect covered by a split-thickness skin graft, taken 2-days after local muscle flap coverage.

On the second postoperative day, after ensuring flap viability, a split-thickness skin graft was harvested from the thigh and applied over the muscle flaps (Figure 10).

Three weeks later, partial weight-bearing was initiated. The bone transition process, at a rate of 1 mm/day, took approximately 110 days to cover the 11-centimeter gap. The total transport time, including the initial healing period, was 117 days (17 weeks). After complete transition, the patient was allowed to bear full weight.

**Figure 11:**
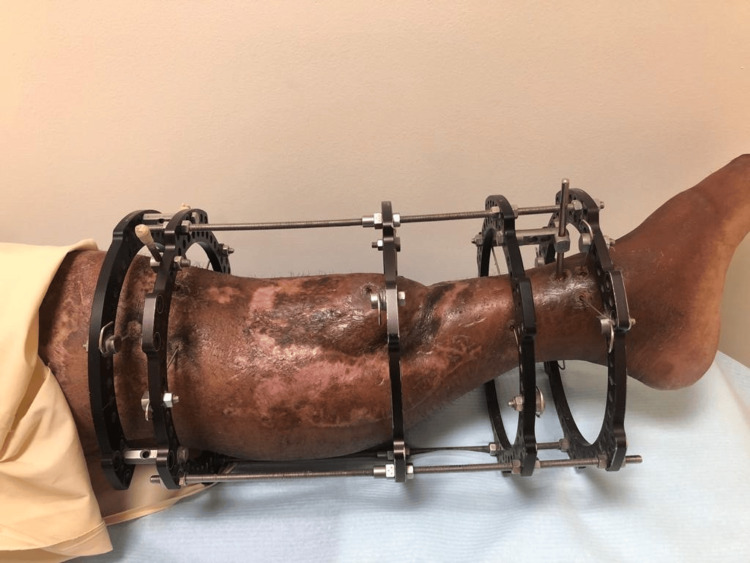
Healed skin flap and graft at the 6 month follow-up appointment

The regenerated bone required another 17 weeks to mature. The external fixation frame was then gradually adjusted (dynamized) over four weeks to prepare for removal. The frame was finally removed after 38 weeks. The patient underwent regular follow-up visits until complete wound healing (Figure 11). The flap remained viable, and no further surgical interventions were necessary. The patient regained full weight-bearing capacity, achieved complete independence, and returned to normal daily activities by the end of the follow-up period.

## Discussion

Traditionally, large soft tissue defects in the lower leg have been resurfaced with free flaps. However, due to extensive soft tissue injury and the involvement of vessels within the injury zone, combined with a lack of reliable recipient vessels, it can be challenging to use free flaps for wound resurfacing. Reconstruction with large flaps and long pedicles or even venous loops often requires microsurgical expertise and prolonged operating times. The combined hemigastrocnemius and hemisoleus muscle flap approach offers several advantages, including the elimination of the need for microsurgery, simplifying and accelerating the operation, reducing costs, and providing the same quality and cosmetic results as soft-tissue coverage for extensive tibial wounds [10-16].

The combined hemigastrocnemius and hemisoleus muscle flap was first introduced by Hyodo in 2004 [10]. Originally limited to a single pedicle, its adaptability has been increased by later methods. Studies have shown no significant functional deficits associated with using this flap, and it can effectively cover large soft tissue defects in the lower leg [10-17].

In this particular case, the patient presented with a severe open fracture of the tibia and fibula, complicated by a vascular injury. The extensive soft tissue damage and popliteal artery involvement made traditional free flap reconstruction challenging. The combined hemigastrocnemius and hemisoleus muscle flap was chosen as a viable alternative due to its ability to provide adequate coverage while minimizing the need for complex microsurgical techniques.

While the combined hemigastrocnemius and hemisoleus muscle flap offers many advantages, it has limitations. One major concern is the restricted reach of the flap, which may not provide sufficient coverage for defects extending beyond the proximal or mid-third of the tibia. This limitation arises due to the anatomical constraints of the flap's pedicles, making it unsuitable for more distal injuries. Additionally, in cases of extensive soft tissue damage or infection, the quality and viability of the local musculature can be compromised, reducing the success rate of this approach. This underscores the importance of careful patient selection and thorough preoperative assessment to determine the technique's feasibility.

Another potential drawback is the risk of donor site morbidity. Harvesting the hemigastrocnemius and hemisoleus muscles may result in residual weakness or compromised function in the lower leg, despite studies suggesting minimal functional loss. This can impact patients who require significant strength or endurance in their calf muscles for daily activities or occupational demands. Furthermore, while eliminating microsurgery simplifies the procedure, it also limits the versatility in addressing larger or more complex defects that might benefit from free flap reconstruction. These considerations highlight the need for individualized surgical planning and a multidisciplinary approach to optimize outcomes in challenging cases.

Preoperative CT angiography played a crucial role in planning the surgical approach. The imaging study identified intact sural artery branches from the popliteal artery by visualizing the blood flow dynamics in the lower extremity. This information was essential in determining the feasibility of using a local muscle flap for soft tissue coverage, ultimately facilitating a less invasive and more efficient surgical procedure.

We propose that preoperative CT angiography be performed in such surgeries to verify the integrity of the sural artery. Additionally, intraoperative muscle exploration should be conducted to exclude any potential muscle trauma. These two steps are essential for the effective planning of local muscle flaps, leading to optimal and satisfactory outcomes and broadening the applicability of this technique in complex cases.

The flap was successfully raised and transferred to the defect, providing adequate coverage for the exposed bone and soft tissue. Despite the initial challenges posed by the vascular injury, the patient ultimately achieved complete wound healing and functional recovery. This case highlights the versatility and effectiveness of the combined hemigastrocnemius and hemisoleus muscle flap in managing complex lower extremity injuries, particularly those involving extensive soft tissue defects and vascular compromise.

The patient underwent regular follow-up visits until the wound healed completely. The flap remained viable, and no further surgical interventions were necessary. The patient regained partial weight-bearing capacity at 3 weeks and full weight-bearing capacity at 17 weeks, along with complete independence, and returned to normal daily activities by the end of the 17 weeks.

## Conclusions

This case underscores the efficacy and versatility of the combined hemigastrocnemius and hemisoleus muscle flap as a reliable alternative for managing complex lower extremity injuries, particularly in the presence of extensive soft tissue defects and vascular compromise. This approach simplifies the reconstruction process by eliminating the need for microsurgical techniques while maintaining functional and cosmetic outcomes. Preoperative CT angiography and meticulous intraoperative planning proved critical in ensuring the success of this technique, emphasizing their importance in similar cases. The patient's complete recovery and return to normal activities highlight the potential of this method to achieve favorable outcomes in challenging clinical scenarios, thus expanding the scope of limb salvage in orthopedic and reconstructive surgery.
